# The genome sequence of the high brown fritillary, *Fabriciana adippe* (Dennis & Schiffermüller, 1775)

**DOI:** 10.12688/wellcomeopenres.18569.1

**Published:** 2022-12-09

**Authors:** Konrad Lohse, Roger Vila, Alex Hayward, Dominik R Laetsch, Wellcome Sanger Institute Tree of Life programme, Wellcome Sanger Institute Scientific Operations: DNA Pipelines collective, Tree of Life Core Informatics collective, Niklas Wahlberg, Darwin Tree of Life Consortium

**Affiliations:** 1 Institute of Ecology and Evolution, University of Edinburgh, Edinburgh, UK; 2 Institut de Biologia Evolutiva (CSIC - Universitat Pompeu Fabra), Barcelona, Spain; 3 College of Life and Environmental Sciences, Department of Biosciences, University of Exeter, Exeter, UK; 4 Department of Biology, Lund University, Lund, Sweden

**Keywords:** Fabriciana adippe, high brown fritillary, genome sequence, chromosomal, Lepidoptera

## Abstract

We present a genome assembly from an individual female *Fabriciana adippe* (the high brown fritillary; Arthropoda; Insecta; Lepidoptera; Nymphalidae). The genome sequence is 485 megabases in span. Most of the assembly (99.98%) is scaffolded into 29 chromosomal pseudomolecules with the Z sex chromosome assembled. The complete mitochondrial genome was also assembled and is 15.1 kilobases in length. Gene annotation of this assembly in Ensembl identified 13,536 protein coding genes.

## Species taxonomy

Eukaryota; Metazoa; Ecdysozoa; Arthropoda; Hexapoda; Insecta; Pterygota; Neoptera; Endopterygota; Lepidoptera; Glossata; Ditrysia; Papilionoidea; Nymphalidae; Heliconiinae; Argynnini; *Fabriciana*; *Fabriciana adippe* (Dennis & Schiffermüller, 1775) (NCBI:txid405009).

## Background

The high brown fritillary, *Fabriciana adippe* (Dennis and Schiffermüller, 1775), is a widespread and common species in Eurasia, but is one of Britain’s most endangered butterflies ( Fox *et al.*, 2015). It is found in open habitats such as forest clearings, which are often covered in bracken. *F. adippe* is a univoltine summer species that can be seen on the wing from May to the beginning of September. In the UK, larval host plants are violets ( *Viola* sp.), for example the common dog-violet ( *Viola riviniana*). The species was placed in the genus *Argynnis* in the past, but recent molecular work suggests that *F. adippe* and allies are more closely related to the genus *Speyeria* than to *Argynnis*, hence its placement in the genus *Fabriciana* ( de Moya *et al.*, 2017).

While *F. adippe* is considered a species of Least Concern according to the IUCN Red List for Europe ( van Swaay *et al.*, 2010), it is listed as endangered on the UK Red List ( Fox *et al.*, 2022), with only 37 populations remaining in the UK ( Ellis *et al.*, 2019). Decreased coppicing and local conservation efforts to implement changes in forest management have enjoyed some success in protecting this species ( Ellis *et al.*, 2019). Today, *F. adippe* is restricted to a few localities in western England and Wales. It is a large, fast flying species, and can be confused with several other similar sized species, including the dark green fritillary ( *Speyeria aglaja*) and the niobe fritillary ( *F. niobe*).

*Fabriciana adippe* has 29 chromosome pairs and a ZO sex-determination system ( Federley, 1938; Lorković, 1941). The genome sequencing of the high brown fritillary may help in conservation efforts and in understanding its population structure, which is notably marked. In fact, Iberian populations are strongly differentiated from other populations in both nuclear and mitochondrial genomes, and admixed populations seem to exist at the contact zone ( Dapporto *et al.*, 2022; Polic *et al.*, 2022).

## Genome sequence report

**Figure 1.**
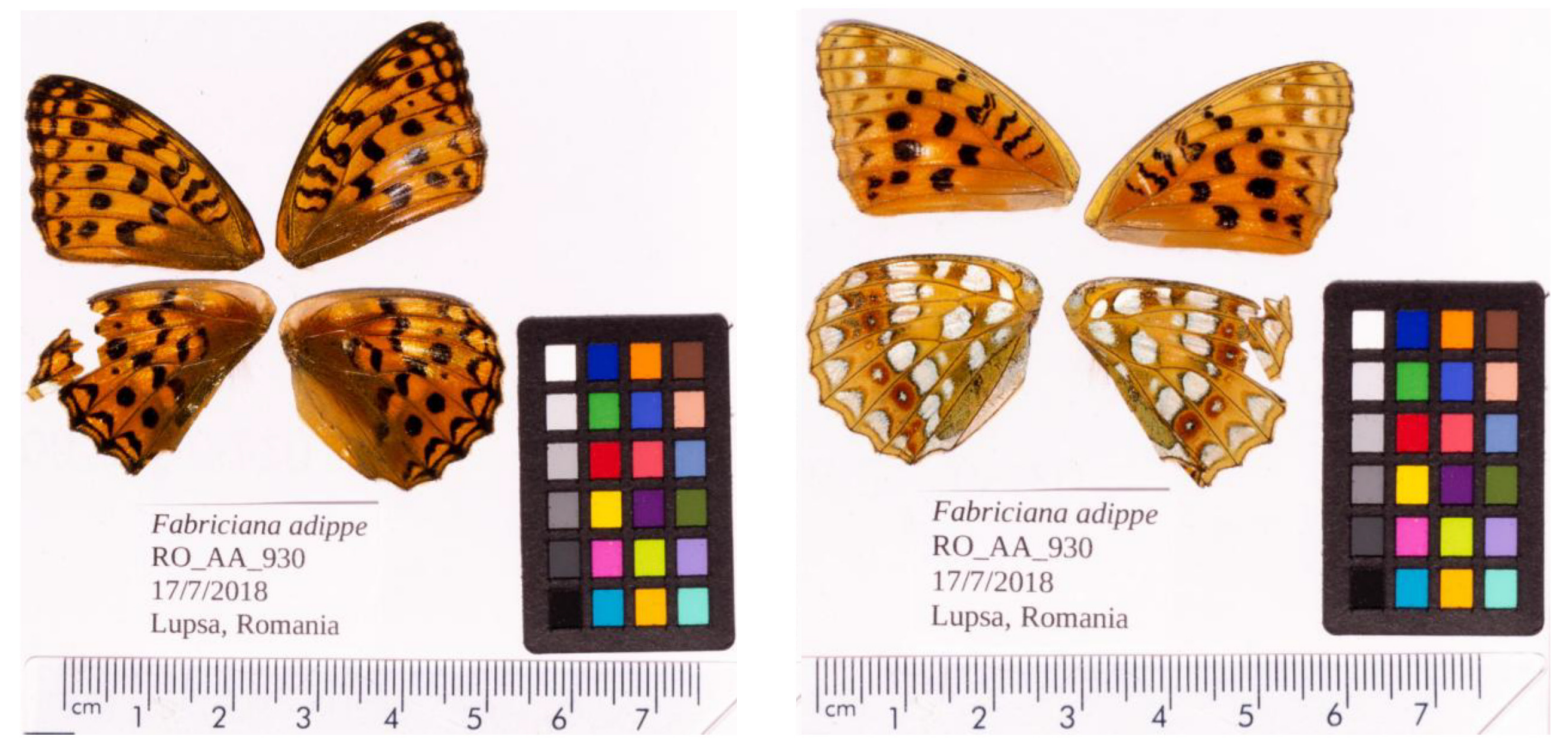
Forewings and hindwings of the female *F. adippe* specimen from which the genome was sequenced. Dorsal (left) and ventral (right) surface view of wings from specimen RO_FA_930 (ilFabAdip1) from Lupşa, Alba, Romania, used to generate Pacific Biosciences, 10X genomics and Hi-C data.

The genome was sequenced from a single female *F. adippe* collected from Lupşa, Apuseni Mountains, Alba, Romania ( Figure 1). A total of 49-fold coverage in Pacific Biosciences single-molecule HiFi long reads and 79-fold coverage in 10X Genomics read clouds were generated. Primary assembly contigs were scaffolded with chromosome conformation Hi-C data. Manual assembly curation corrected 26 missing/misjoins and removed six haplotypic duplications, reducing the assembly size by 1.90% and the scaffold number by 9.62%.

**Figure 2.**
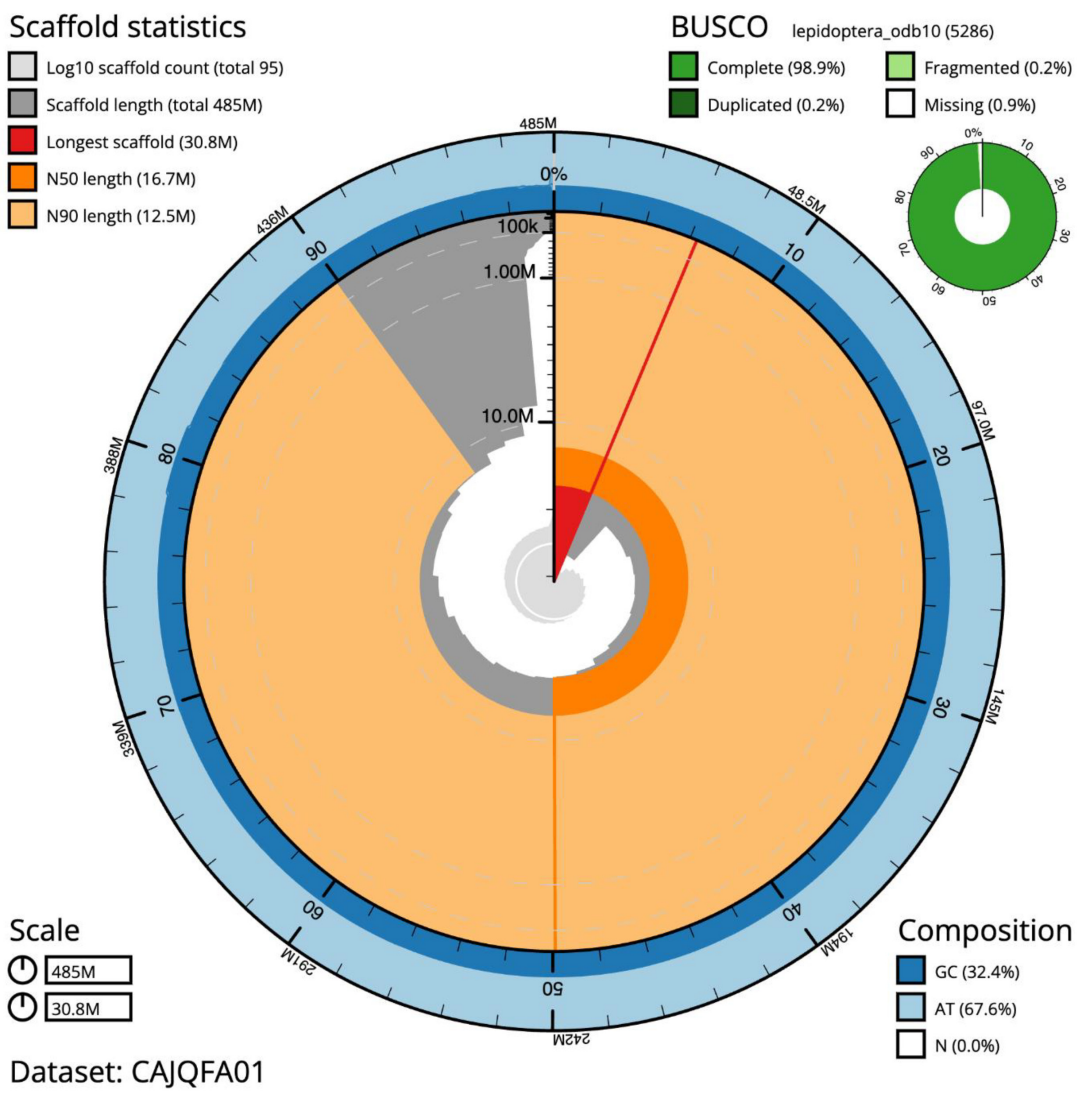
Genome assembly of *F. adippe*, ilFabAdip1.1: metrics. The BlobToolKit Snailplot shows N50 metrics and BUSCO gene completeness. The main plot is divided into 1,000 size-ordered bins around the circumference with each bin representing 0.1% of the 484,826,487 bp assembly. The distribution of chromosome lengths is shown in dark grey with the plot radius scaled to the longest chromosome present in the assembly (30,838,137 bp, shown in red). Orange and pale-orange arcs show the N50 and N90 chromosome lengths (16,739,368 and 12,513,400 bp), respectively. The pale grey spiral shows the cumulative chromosome count on a log scale with white scale lines showing successive orders of magnitude. The blue and pale-blue area around the outside of the plot shows the distribution of GC, AT and N percentages in the same bins as the inner plot. A summary of complete, fragmented, duplicated and missing BUSCO genes in the lepidoptera_odb10 set is shown in the top right. An interactive version of this figure is available at https://blobtoolkit.genomehubs.org/view/ilFabAdip1.1/dataset/CAJQFA01/snail.

The final assembly has a total length of 485 Mb in 94 sequence scaffolds with a scaffold N50 of 16.7 Mb ( Table 1). Most of the assembly sequence (99.98%) was assigned to 29 chromosomal-level scaffolds, representing 28 autosomes (numbered by sequence length) and the Z sex chromosome ( Figure 2– Figure 5; Table 2).

**Table 1.** Genome data for *F. adippe*, ilFabAdip1.1.

*Project accession data*	
Assembly identifier	ilFabAdip1.1
Species	*Fabriciana adippe*
Specimen	ilFabAdip1 (genome assembly, Hi-C); ilFabAdip2 (RNA-Seq)
NCBI taxonomy ID	405009
BioProject	PRJEB43797
BioSample ID	SAMEA7523308
Isolate information	ilFabAdip1: female, abdomen tissue (genome assembly), thorax tissue (Hi-C). ilFabAdip2: male, abdomen tissue (RNA-Seq).
*Raw data accessions*	
PacificBiosciences SEQUEL II	ERR6565939
10X Genomics Illumina	ERR6054608-ERR6054611
Hi-C Illumina	ERR6054612
PolyA RNA-Seq Illumina	ERR6054613
*Genome assembly*	
Assembly accession	GCA_905404265.1
*Accession of alternate * *haplotype*	GCA_905404255.1
Span (Mb)	485
Number of contigs	110
Contig N50 length (Mb)	15.9
Number of scaffolds	94
Scaffold N50 length (Mb)	16.7
Longest scaffold (Mb)	26.1
BUSCO * genome score	C:98.9%[S:98.8%,D:0.2%],F:0.2%,M:0.9%,n:5,286
*Genome annotation*	
Number of protein-coding genes	13,536
Number of noncoding genes	8,725

*BUSCO scores based on the lepidoptera_odb10 BUSCO set using v5.3.2. C = complete [S = single copy, D = duplicated], F = fragmented, M = missing, n = number of orthologues in comparison. A full set of BUSCO scores is available at https://blobtoolkit.genomehubs.org/view/ilFabAdip1.1/dataset/CAJQFA01/busco.

**Figure 3.**
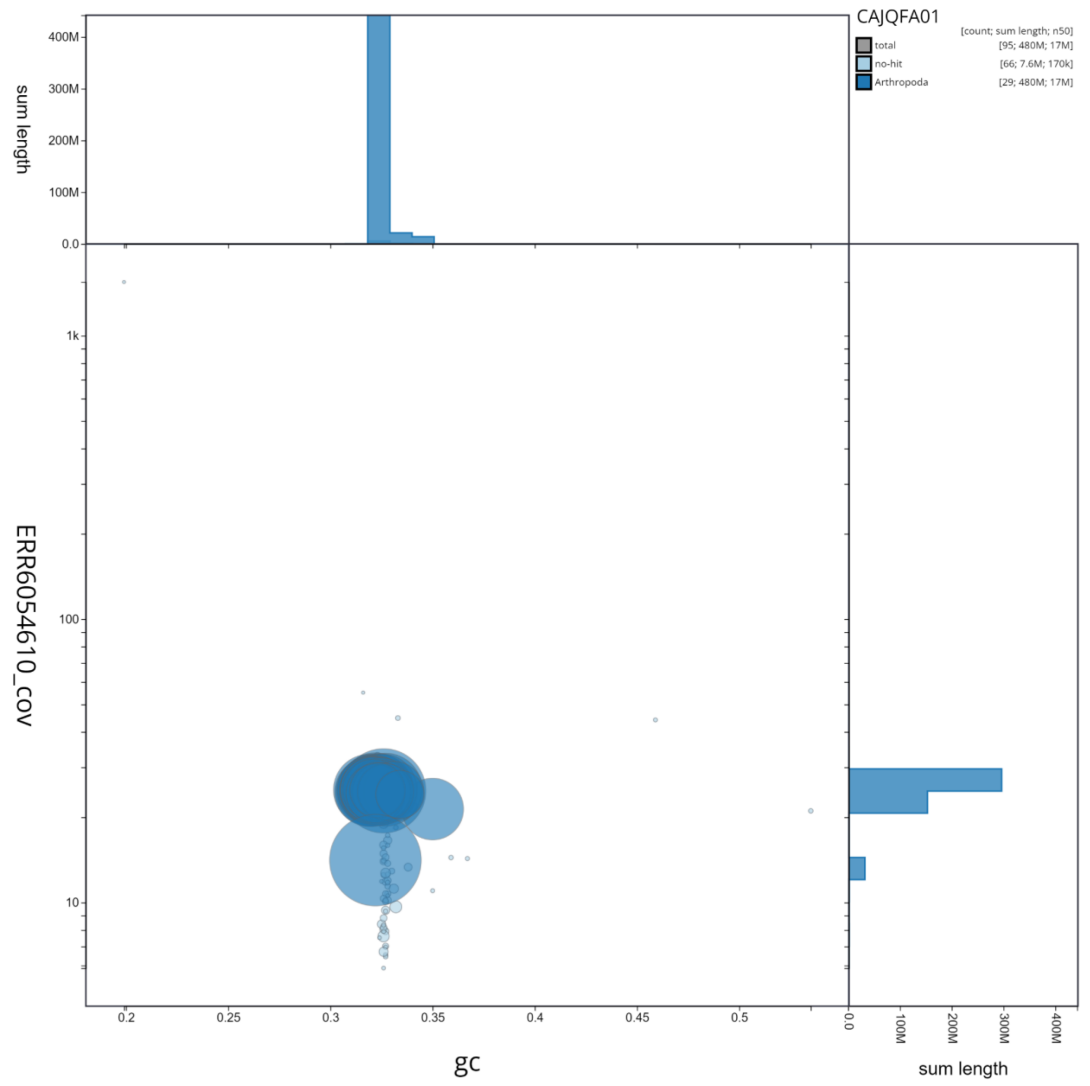
Genome assembly of *F. adippe*, ilFabAdip1.1: GC coverage. BlobToolKit GC-coverage plot. Scaffolds are coloured by phylum. Circles are sized in proportion to scaffold length. Histograms show the distribution of scaffold length sum along each axis. An interactive version of this figure is available at https://blobtoolkit.genomehubs.org/view/ilFabAdip1.1/dataset/CAJQFA01/blob.

**Figure 4.**
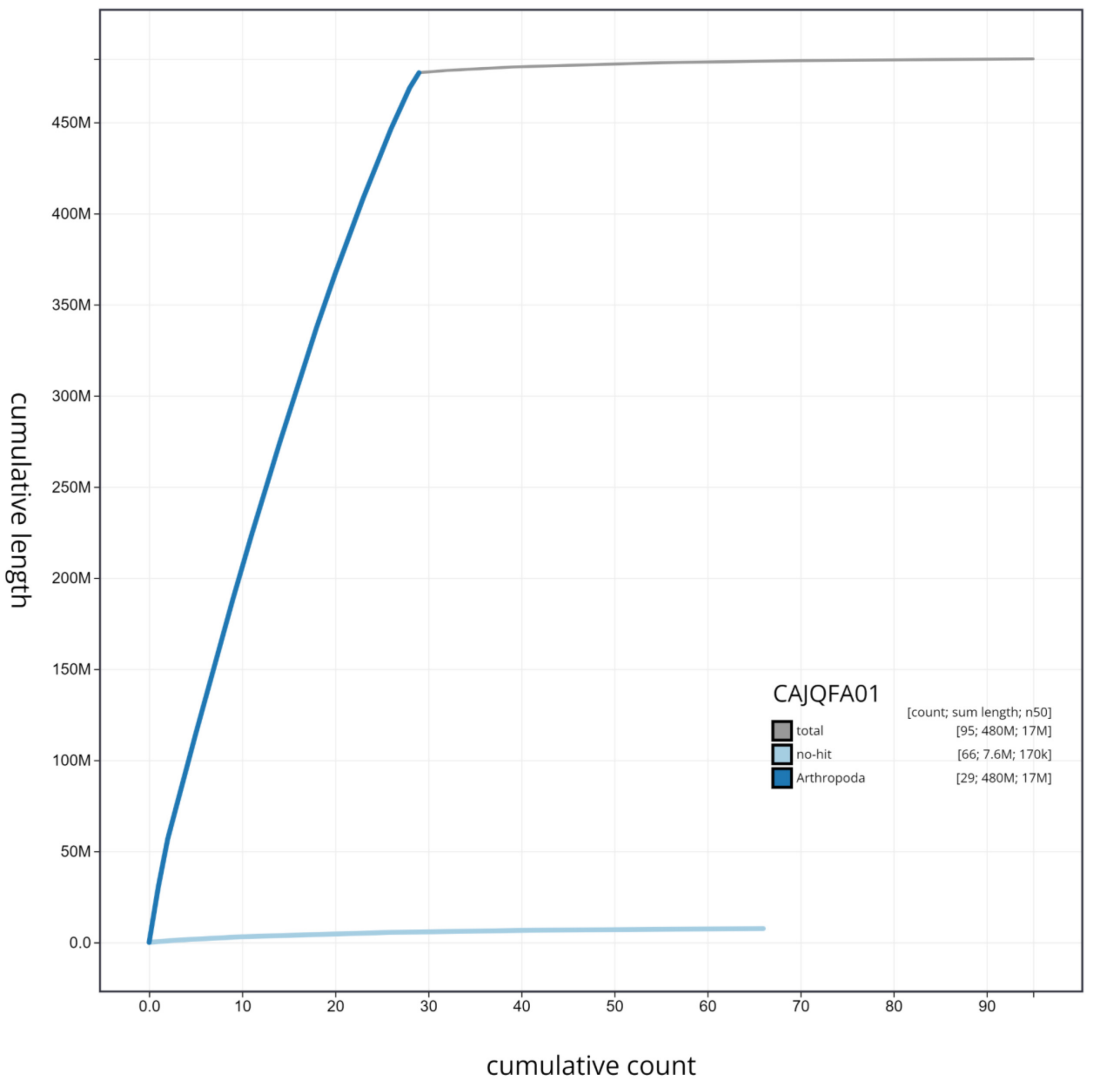
Genome assembly of *F. adippe*, ilFabAdip1.1: cumulative sequence. BlobToolKit cumulative sequence plot. The grey line shows cumulative length for all scaffolds. Coloured lines show cumulative lengths of scaffolds assigned to each phylum using the buscogenes taxrule. An interactive version of this figure is available at https://blobtoolkit.genomehubs.org/view/ilFabAdip1.1/dataset/CAJQFA01/cumulative.

**Figure 5.**
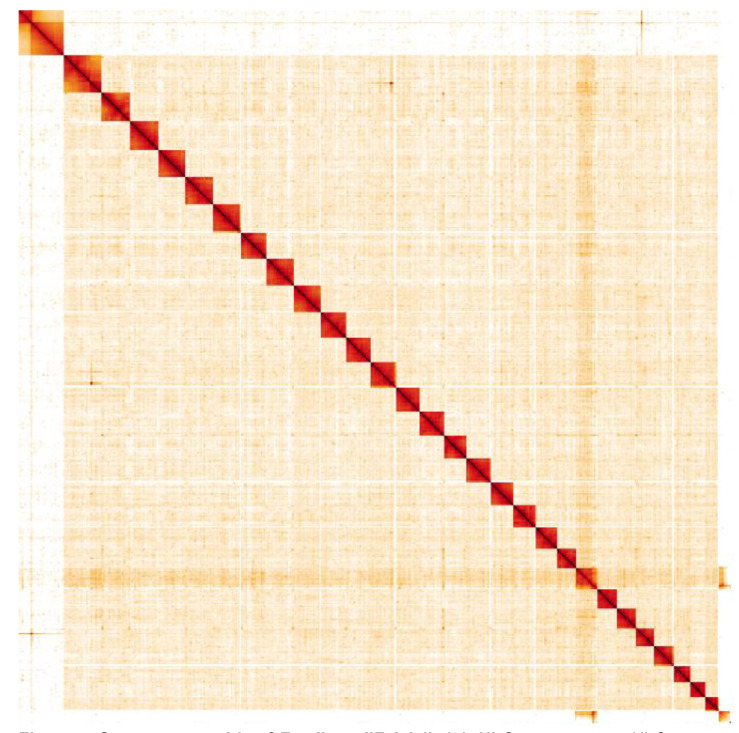
Genome assembly of *F. adippe*, ilFabAdip1.1: Hi-C contact map. Hi-C contact map of the ilFabAdip1.1 assembly, visualised in HiGlass. Chromosomes are arranged in size order from left to right and top to bottom. The interactive Hi-C map can be viewed at https://genome-note-higlass.tol.sanger.ac.uk/l/?d=G2lH9zghRgeH6ugDELRW7g.

**Table 2.** Chromosomal pseudomolecules in the genome assembly of *F. adippe*, ilFabAdip1.1.

INSDC accession	Chromosome	Size (Mb)	GC%
FR989983.1	1	26.05	32.6
FR990003.1	2	13.83	35
FR989984.1	3	19.37	32.7
FR989985.1	4	19.29	32.2
FR989986.1	5	18.82	32.3
FR989987.1	6	18.7	31.9
FR989988.1	7	18.55	32.1
FR989989.1	8	18.33	32.5
FR989990.1	9	18.32	32.1
FR989991.1	10	17.98	32
FR989992.1	11	17.21	31.9
FR989993.1	12	16.91	32
FR989994.1	13	16.74	32.1
FR989995.1	14	16.56	32.1
FR989996.1	15	16.42	32.4
FR989997.1	16	15.98	32.1
FR989998.1	17	15.9	32.3
FR989999.1	18	15.65	32.1
FR990000.1	19	14.94	32.5
FR990001.1	20	14.47	32.5
FR990002.1	21	13.83	32.4
FR990004.1	22	13.75	32.1
FR990005.1	23	13.08	33.1
FR990006.1	24	12.55	32.5
FR990007.1	25	12.51	32.2
FR990008.1	26	11.41	32.7
FR990009.1	27	10.96	32.3
FR990010.1	28	8.35	33.4
FR989982.1	Z	30.84	32.2
FR990011.1	MT	0.02	19.4
-	Unplaced	7.56	33

The assembly has a BUSCO v5.3.2 ( Manni *et al.*, 2021) completeness of 98.9% (single 98.8%, duplicated 0.1%) using the lepidoptera_odb10 reference set ( *n* = 5,286). While not fully phased, the assembly deposited is of one haplotype. Contigs corresponding to the second haplotype have also been deposited.

## Genome annotation report

The *F. adippe* genome was annotated using the Ensembl rapid annotation pipeline ( Table 1; https://rapid.ensembl.org/Fabriciana_adippe_GCA_905404265.1/). The resulting annotation includes 35,064 transcribed mRNAs from 13,536 protein-coding and 8,725 non-coding genes.

## Methods

### Sample acquisition and nucleic acid extraction

Two *F. adippe* specimens (ilFabAdip1, female, genome assembly and Hi-C; ilFabAdip2, male, RNA-Seq) were collected using a handnet in Lupşa, Apuseni Mountains, Alba, Romania (latitude 46.416, longitude 23.192) by Konrad Lohse, Dominik Laetsch (University of Edinburgh), Alex Hayward (University of Exeter) and Roger Vila (Institut de Biologia Evolutiva, Barcelona). The specimens were identified by Roger Vila and snap-frozen from live in a dry shipper.

DNA was extracted at the Scientific Operations Core, Wellcome Sanger Institute. The ilFabAdip1 sample was weighed and dissected on dry ice with thorax tissue set aside for Hi-C sequencing. Abdomen tissue was disrupted by manual grinding with a disposable pestle. Fragment size analysis of 0.01–0.5 ng of DNA was then performed using an Agilent FemtoPulse. High molecular weight (HMW) DNA was extracted using the Qiagen MagAttract HMW DNA extraction kit. Low molecular weight DNA was removed from a 200 ng aliquot of extracted DNA using 0.8X AMpure XP purification kit prior to 10X Chromium sequencing. A minimum of 50 ng DNA was submitted for 10X sequencing. HMW DNA was sheared into an average fragment size of 12–20 kb in a Megaruptor 3 system with speed setting 30. Sheared DNA was purified by solid-phase reversible immobilisation using AMPure PB beads with a 1.8X ratio of beads to sample to remove the shorter fragments and concentrate the DNA. The concentration of the sheared and purified DNA was assessed using a Nanodrop spectrophotometer and Qubit Fluorometer and Qubit dsDNA High Sensitivity Assay kit. Fragment size distribution was evaluated by running the sample on the FemtoPulse system.

RNA was extracted from abdomen tissue of ilFabAdip2 in the Tree of Life Laboratory at the WSI using TRIzol, according to the manufacturer’s instructions. RNA was then eluted in 50 μl RNAse-free water and its concentration RNA assessed using a Nanodrop spectrophotometer and Qubit Fluorometer using the Qubit RNA Broad-Range (BR) Assay kit. Analysis of the integrity of the RNA was done using Agilent RNA 6000 Pico Kit and Eukaryotic Total RNA assay.

### Sequencing

Pacific Biosciences HiFi circular consensus and 10X Genomics Chromium read cloud sequencing libraries were constructed according to the manufacturers’ instructions. Sequencing was performed by the Scientific Operations core at the Wellcome Sanger Institute on Pacific Biosciences SEQUEL II (HiFi), Illumina HiSeq 10X and Illumina HiSeq 4000 (RNA-Seq) instruments. Hi-C data were generated in the Tree of Life laboratory from thorax tissue of ilFabAdip1 using the Arima v2 kit and sequenced on a HiSeq 10X instrument.

### Genome assembly

Assembly was carried out with Hifiasm ( Cheng *et al.*, 2021); haplotypic duplication was identified and removed with purge_dups ( Guan *et al.*, 2020). One round of polishing was performed by aligning 10X Genomics read data to the assembly with longranger align, calling variants with freebayes ( Garrison & Marth, 2012). The assembly was then scaffolded with Hi-C data ( Rao *et al.*, 2014) using SALSA2 ( Ghurye *et al.*, 2019). The assembly was checked for contamination and corrected using the gEVAL system ( Chow *et al.*, 2016) as described previously ( Howe *et al.*, 2021). Manual curation ( Howe *et al.*, 2021) was performed using gEVAL, HiGlass ( Kerpedjiev *et al.*, 2018) and Pretext ( Harry, 2022). The mitochondrial genome was assembled using MitoHiFi ( Uliano-Silva *et al.*, 2021), which performs annotation using MitoFinder ( Allio *et al.*, 2020). The genome was analysed and BUSCO scores generated within the BlobToolKit environment ( Challis *et al.*, 2020). Table 3 contains a list of all software tool versions used, where appropriate.

**Table 3.** Software tools used.

Software tool	Version	Source
BlobToolKit	3.2.6	Challis *et al.*, 2020
freebayes	1.3.1-17- gaa2ace8	Garrison & Marth, 2012
gEVAL	N/A	Chow *et al.*, 2016
Hifiasm	0.12	Cheng *et al.*, 2021
HiGlass	1.11.6	Kerpedjiev *et al.*, 2018
longranger align	2.2.2	https:// support.10xgenomics. com/genome-exome/ software/pipelines/latest/advanced/other- pipelines
MitoHiFi	1.0	Uliano-Silva *et al.*, 2021
PretextView	0.2.x	https://github.com/wtsi- hpag/PretextView
purge_dups	1.2.3	Guan *et al.*, 2020
SALSA2	2.2	Ghurye *et al.*, 2019

### Genome annotation

The Ensembl gene annotation system ( Aken *et al.*, 2016) was used to generate annotation for the *F. adippe* assembly (GCA_905404265.1). Annotation was created primarily through alignment of transcriptomic data to the genome, with gap filling via protein-to-genome alignments of a select set of proteins from UniProt ( UniProt Consortium, 2019).

## Data Availability

European Nucleotide Archive: *Fabriciana adippe* (high brown fritillary). Accession number PRJEB43797; https://identifiers.org/ena.embl/PRJEB43797 ( Wellcome Sanger Institute, 2022) The genome sequence is released openly for reuse. The *F. adippe* genome sequencing initiative is part of the Darwin Tree of Life (DToL) project. All raw sequence data and the assembly have been deposited in INSDC databases. Raw data and assembly accession identifiers are reported in Table 1.

## Author information

Members of the Wellcome Sanger Institute Tree of Life programme are listed here: https://doi.org/10.5281/zenodo.4783585.

Members of Wellcome Sanger Institute Scientific Operations: DNA Pipelines collective are listed here: https://doi.org/10.5281/zenodo.4790455.

Members of the Tree of Life Core Informatics collective are listed here: https://doi.org/10.5281/zenodo.5013541.

Members of the Darwin Tree of Life Consortium are listed here: https://doi.org/10.5281/zenodo.4783558.

## References

[ref-1] Aken BL Ayling S Barrell D : The Ensembl gene annotation system. *Database (Oxford).* 2016;2016:baw093. 10.1093/database/baw093 27337980 PMC4919035

[ref-2] Allio R Schomaker-Bastos A Romiguier J : MitoFinder: Efficient automated large‐scale extraction of mitogenomic data in target enrichment phylogenomics. *Mol Ecol Resour.* 2020;20(4):892–905. 10.1111/1755-0998.13160 32243090 PMC7497042

[ref-3] Challis R Richards E Rajan J : BlobToolKit - interactive quality assessment of genome assemblies. *G3 (Bethesda).* 2020;10(4):1361–1374. 10.1534/g3.119.400908 32071071 PMC7144090

[ref-4] Cheng H Concepcion GT Feng X : Haplotype-resolved *de novo* assembly using phased assembly graphs with hifiasm. *Nat Methods.* 2021;18(2):170–175. 10.1038/s41592-020-01056-5 33526886 PMC7961889

[ref-5] Chow W Brugger K Caccamo M : gEVAL - a web-based browser for evaluating genome assemblies. *Bioinformatics.* 2016;32(16):2508–2510. 10.1093/bioinformatics/btw159 27153597 PMC4978925

[ref-6] Dapporto L Menchetti M Vodă R : The atlas of mitochondrial genetic diversity for Western Palaearctic butterflies. *Global Ecology and Biogeography.* 2022;31(11):2184–2190. 10.1111/geb.13579

[ref-19] de Moya RS Savage WK Tenney C : Interrelationships and diversification of *Argynnis* fabricius and *Speyeria* scudder butterflies. *Systematic Entomology.* 2017;42(4):635–649. 10.1111/syen.12236

[ref-7] Ellis S Wainwright D Dennis EB : Are habitat changes driving the decline of the UK's most threatened butterfly: the High Brown Fritillary *Argynnis adippe* (Lepidoptera: Nymphalidae)? *J Insect Conserv.* 2019;23(2):351–367. 10.1007/s10841-019-00134-0

[ref-8] Federley H : Chromosomenzahlen Finnlän-Discher Lepidopteren: I. Rhopalocera. *Hereditas.* 1938;24(4):397–464. 10.1111/j.1601-5223.1938.tb03219.x

[ref-9] Fox R Brereton TM Asher J : The State of the UK’s Butterflies 2015.Wareham, Dorset: Butterfly Conservation and the Centre for Ecology & Hydrology,2015; (Accessed: 25 October 2022). Reference Source

[ref-10] Fox R Dennis EB Brown AF : A revised Red List of British butterflies. *Insect Conservation and Diversity.* 2022;15(5):485–495. 10.1111/icad.12582

[ref-11] Garrison E Marth G : Haplotype-based variant detection from short-read sequencing. 2012. 10.48550/arXiv.1207.3907

[ref-12] Ghurye J Rhie A Walenz BP : Integrating Hi-C links with assembly graphs for chromosome-scale assembly. *PLoS Comput Biol.* 2019;15(8):e1007273. 10.1371/journal.pcbi.1007273 31433799 PMC6719893

[ref-13] Guan D McCarthy SA Wood J : Identifying and removing haplotypic duplication in primary genome assemblies. *Bioinformatics.* 2020;36(9):2896–2898. 10.1093/bioinformatics/btaa025 31971576 PMC7203741

[ref-14] Harry E : PretextView (Paired REad TEXTure Viewer): A desktop application for viewing pretext contact maps.2022; (Accessed: 19 October 2022). Reference Source

[ref-15] Howe K Chow W Collins J : Significantly improving the quality of genome assemblies through curation. *GigaScience.* Oxford University Press,2021;10(1):giaa153. 10.1093/gigascience/giaa153 33420778 PMC7794651

[ref-16] Kerpedjiev P Abdennur N Lekschas F : HiGlass: Web-based visual exploration and analysis of genome interaction maps. *Genome Biol.* 2018;19(1):125. 10.1186/s13059-018-1486-1 30143029 PMC6109259

[ref-17] Lorković Z : Die Chromosomenzahlen in Der Spermatogenese Der Tagfalter. *Chromosoma.* 1941;2(1):155–191. 10.1007/BF00325958

[ref-18] Manni M Berkeley MR Seppey M : BUSCO Update: Novel and Streamlined Workflows along with Broader and Deeper Phylogenetic Coverage for Scoring of Eukaryotic, Prokaryotic, and Viral Genomes. *Mol Biol Evol.* 2021;38(10):4647–4654. 10.1093/molbev/msab199 34320186 PMC8476166

[ref-20] Polic D Yıldırım Y Lee KM : Linking large-scale genetic structure of three Argynnini butterfly species to geography and environment. *Mol Ecol.* 2022;31(16):4381–4401. 10.1111/mec.16594 35841126 PMC9544544

[ref-21] Rao SS Huntley MH Durand NC : A 3D map of the human genome at kilobase resolution reveals principles of chromatin looping. *Cell.* 2014;159(7):1665–1680. 10.1016/j.cell.2014.11.021 25497547 PMC5635824

[ref-23] Uliano-Silva M : MitoHiFi. 2021; (Accessed: 19 October 2022). Reference Source

[ref-24] UniProt Consortium: UniProt: a worldwide hub of protein knowledge. *Nucleic Acids Res.* 2019;47(D1):D506–D515. 10.1093/nar/gky1049 30395287 PMC6323992

[ref-22] van Swaay C Cuttelod A Collins S : European Red List of Butterflies.IUCN (International Union for Conservation of Nature) and Butterfly Conservation Europe in collaboration with the European Union,2010. 10.2779/83897

[ref-25] Wellcome Sanger Institute: The genome sequence of the high brown fritillary, Fabriciana adippe (Dennis & Schiffermüller, 1775), European Nucleotide Archive.[dataset]. accession number PRJEB43797.2022. 10.12688/wellcomeopenres.18569.1 PMC10186063 37224319

