# Supplementary material for: The genome sequence of the high brown fritillary, Fabriciana adippe (Dennis & Schiffermüller, 1775)
Source: Wellcome Open Res. 2022 Dec 9;7:298. [Version 1] doi: 10.12688/wellcomeopenres.18569.1 (PMC10186063; doi:10.12688/wellcomeopenres.18569.1)
Supplement: Peer review history

# Peer review: Reviewer response for version 1

**DOI:** 10.21956/wellcomeopenres.20590.r57456

**Published:** 2023-05-25

**Authors:** Benoit Nabholz

**Affiliations:** 1 Universite de Montpellier, Montpellier, Occitanie, France

The authors present a chromosome scale assembly of *Fabriciana adippe*. Once again, it is a very nice genomic resource produced by the DToL project. The analyses are the same as in the other genomes of the project.

 As many other butterflies, it is not a sample from the UK, which seems to be at odds with the philosophy of the project. Anyway, the assembly is of high quality and I have nothing to say about the report.

 All the links and the datasets presented are publicly available.

Are sufficient details of methods and materials provided to allow replication by others?

Yes

Is the rationale for creating the dataset(s) clearly described?

Yes

Are the datasets clearly presented in a useable and accessible format?

Yes

Are the protocols appropriate and is the work technically sound?

Yes

Reviewer Expertise:

Evolutionary biology, Population genomic

I confirm that I have read this submission and believe that I have an appropriate level of expertise to confirm that it is of an acceptable scientific standard.

# Peer review: Reviewer response for version 1

**DOI:** 10.21956/wellcomeopenres.20590.r56810

**Published:** 2023-05-15

**Authors:** Annabel Whibley

**Affiliations:** 1 The University of Auckland, Auckland, Auckland, New Zealand

Lohse and colleagues describe the assembly and annotation of the genome of the high brown fritillary, *Fabriciana adippe. *This high-quality genome, processed through genome sequencing consortia pipelines with clear reporting of tools and versioning, has superb quality statistics and will be a useful genomics resource for the community. Data has been openly released.

 From the abstract, it was unclear to me whether the Z chromosome was part of the "29 chromosomal pseudomolecules" but that there are 28 autosomes plus the Z (with Z/O sex determination) is stated in the body of the report.

 I would be curious to know whether the reduced coverage of the Z in the female genome(i.e. 1 copy vs 2 of the autosomes) had any impact on the quality of the assembly of the sex chromosome. If there is a high level of heterozygosity, then perhaps the lower coverage would be compensated for by the lack of a second copy of this chromosome.

 "Read clouds" in the context of 10X data was a term that I hadn't encountered before, although the meaning is quite intuitive- i.e. the set of reads associated with the same barcode sequences.

 There is a typo in line 5 of the second methods paragraph (AMpure should be AMPure, it is correct in subsequent lines).

Are sufficient details of methods and materials provided to allow replication by others?

Yes

Is the rationale for creating the dataset(s) clearly described?

Yes

Are the datasets clearly presented in a useable and accessible format?

Yes

Are the protocols appropriate and is the work technically sound?

Yes

Reviewer Expertise:

Genomics in plants and animals, including genome assembly.

I confirm that I have read this submission and believe that I have an appropriate level of expertise to confirm that it is of an acceptable scientific standard.

# Peer review: Reviewer response for version 1

**DOI:** 10.21956/wellcomeopenres.20590.r56811

**Published:** 2023-05-02

**Authors:** Arnaud Martin

**Affiliations:** 1 Department of Biological Sciences, The George Washington University, Washington, District of Columbia, USA

This genome report describes a near-complete assembly for the butterfly *Fabriciana addipe, *an important resource for the study of lepidopteran diversity. I was first surprised to see no W chromosome, and then learned in the text that "ZO sex-determination system", so it will be interesting to study this novelty in a phylogenetic framework in the future. 

 Wiemers et al. 2020 could be used to strengthen the placement of this butterfly in a monophyletic *Fabriciana* genus ^1^.

Are sufficient details of methods and materials provided to allow replication by others?

Yes

Is the rationale for creating the dataset(s) clearly described?

Yes

Are the datasets clearly presented in a useable and accessible format?

Yes

Are the protocols appropriate and is the work technically sound?

Yes

Reviewer Expertise:

Butterfly development genetics and evolution

I confirm that I have read this submission and believe that I have an appropriate level of expertise to confirm that it is of an acceptable scientific standard.

## References

[1] : A complete time-calibrated multi-gene phylogeny of the European butterflies. *Zookeys* .2020;938: 10.3897/zookeys.938.50878 97-124 10.3897/zookeys.938.50878 32550787PMC7289901
